# Using machine learning on clinical data to identify unexpected patterns in groups of COVID-19 patients

**DOI:** 10.1038/s41598-022-26294-9

**Published:** 2023-02-08

**Authors:** Hannah Paris Cowley, Michael S. Robinette, Jordan K. Matelsky, Daniel Xenes, Aparajita Kashyap, Nabeela F. Ibrahim, Matthew L. Robinson, Scott Zeger, Brian T. Garibaldi, William Gray-Roncal

**Affiliations:** 1 Research and Exploratory Development Department, The Johns Hopkins University Applied Physics Laboratory, Laurel, MD USA; 2 The Johns Hopkins University School of Medicine, Baltimore, MD USA; 3 The Johns Hopkins University Bloomberg School of Public Health, Baltimore, MD USA

**Keywords:** Diagnostic markers, Predictive markers, Risk factors

## Abstract

As clinicians are faced with a deluge of clinical data, data science can play an important role in highlighting key features driving patient outcomes, aiding in the development of new clinical hypotheses. Insight derived from machine learning can serve as a clinical support tool by connecting care providers with reliable results from big data analysis that identify previously undetected clinical patterns. In this work, we show an example of collaboration between clinicians and data scientists during the COVID-19 pandemic, identifying sub-groups of COVID-19 patients with unanticipated outcomes or who are high-risk for severe disease or death. We apply a random forest classifier model to predict adverse patient outcomes early in the disease course, and we connect our classification results to unsupervised clustering of patient features that may underpin patient risk. The paradigm for using data science for hypothesis generation and clinical decision support, as well as our triaged classification approach and unsupervised clustering methods to determine patient cohorts, are applicable to driving rapid hypothesis generation and iteration in a variety of clinical challenges, including future public health crises.

## Introduction

The integration of machine learning and data science analysis into clinical care represents a burgeoning opportunity to deliver even more impactful patient care^1–8^, enabled in part by the vast amount of complex data that is collected as part of routine clinical encounters^9,10^. Inpatient hospital stays result in large amounts of patient data, including continuous monitoring of vital signs, frequent laboratory tests, clinical observations, radiology images, and more. These varied data modalities and their associated features are collected across a large patient population. While it is challenging for even the most skilled clinician to synthesize and integrate the wealth of health data available into immediate clinical decision-making, these data are particularly valuable for assessing patterns in the population, such as identifying sub-groups of patients afflicted with a given disease. Leveraging big data to improve healthcare at an individualized level is the essence of precision medicine^11^.

Precision medicine and associated big data modeling approaches can be used to augment clinical opinion, especially in the intensive care setting^3,4,8,12,13^. One of the most famous examples of this is the Acute Physiology and Chronic Health Evaluation (APACHE) model that predicts mortality of adult ICU patients using logistic regression with features derived from the first day of a patient’s ICU stay^14^. Additionally, precision medicine tools can be used in a retrospective manner to find important features and patterns in individual patients and patient sub-group trajectories that can be used prospectively in the care of future patients^4,15–20^.

**Figure 1:**
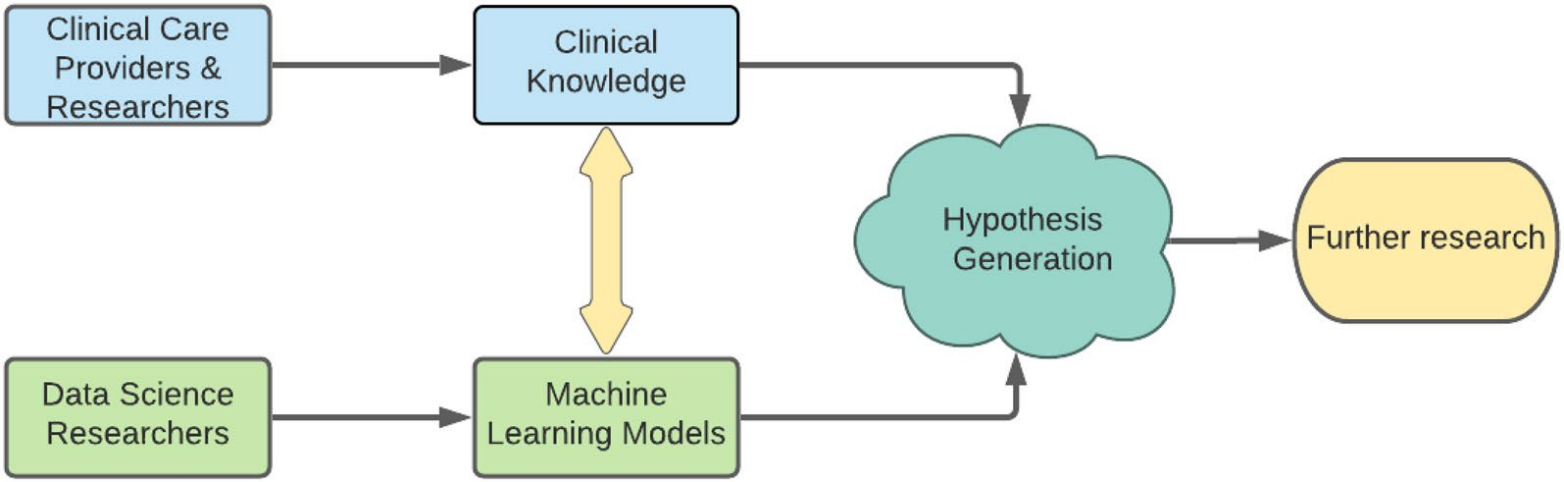
Clinical knowledge and data science modeling work in tandem to drive hypothesis generation. In our approach, we combine machine learning methods with clinical knowledge to generate hypothesized patient sub-groups and factors that affect patient outcomes. The collaboration between clinical insight and machine learning modeling informs the generation of new hypotheses which may then be tested in further research. The yellow arrow indicates the critical interplay between clinical knowledge and machine learning modeling: clinical knowledge informs the construction of models and the results of models are checked against clinical knowledge.

Although precision medicine approaches are often tailored to a specific disease or use-case, the method we describe is a readily generalizable model with a special focus on data exploration for identification of unexpected clinical patterns and development of new avenues for scientific exploration. We apply our model to COVID-19, but emphasize the potential utility of our approach in a variety of diseases—especially those for which timely diagnosis and treatment are critical for promoting patient health. In addition to predicting COVID-19 outcomes and identifying patient sub-groups, an important aspect of this work is the implementation of a generalizable conceptual framework and technical method to combine clinical knowledge and machine learning models. A schematic of this conceptual framework can be found in Fig. 1. These models may be used as hypothesis generation tools, which may be particularly useful in emerging health crises, where established and effective treatment protocols do not exist and where it is preferable to use only low-cost clinical resources (e.g., laboratory blood tests, vital sign measurements). Through analyzing the error patterns of imperfect models and using the modeling approach to identify patterns in patients with unexpected outcomes, we are able to pose potential avenues for further clinical investigation. The use of such methods may spur new thought into the patterns underlying elevated risk of adverse outcomes.

In this paper, we present a two-pronged approach to understanding COVID-19 disease presentation and progression in patient sub-groups. The first is a supervised machine learning algorithm, the “triaged” prediction model, designed to investigate features underlying patient outcomes and highlight patients who may have unexpected outcomes. The triaged prediction approach focuses on two goals: (1) identifying whether a patient will have a mild disease course or develop severe disease and/or death within fourteen days of hospital admission (where severe disease is defined as need for high-flow nasal cannula, non-invasive ventilation, or mechanical ventilation) and (2) identifying the patterns in patient variables responsible for differences in our ability to correctly classify patient outcomes.

A complementary unsupervised machine learning clustering algorithm was built to find subsets of patients who present similarly at time of hospital admission and may represent distinct, clinically significant sub-groups. These analysis strategies utilize clinical input at all stages of research, from data collection to testing hypotheses generated from the machine learning methods. Using the complementary approaches of supervised and unsupervised machine learning, we are able to analyze characteristics underpinning patient sub-group identification and prognosis. This method enables us to explore the heterogeneity of patient presentation and the limits of these machine learning algorithms for identifying patient sub-groups and predicting outcomes for each of these populations.

## Methods

### Data

The data used were part of JH-CROWN: The COVID Precision Medicine Analytics Platform Registry^21^ and some patients analyzed here have been included in previous descriptions of the cohort^8,17–19,22–24^. Data from 1175 patients in JH-CROWN were used in this analysis. This research was reviewed and approved by the Johns Hopkins Medicine Institutional Review Board (IRB) (IRB00250975), and all methods were conducted in accordance with the IRB guidelines and regulations for this style of research. Because this research posed minimal risk to participants and due to the retrospective nature of this work, the study was approved under a waiver of informed consent for participants.

Outcomes of interest in this study were defined as “mild”, hospital discharge without need for more intensive therapy than low-flow oxygen, or “severe/death”, where severe disease is defined as need for high-flow nasal cannula, non-invasive ventilation, or mechanical ventilation. For each patient, data from the first 48 hours after hospital admission were analyzed. The 48 hours were divided into 8 distinct 6-hour intervals, termed “epochs”. Patients were excluded from analysis if they achieved an outcome of interest within the first epoch, or if they did not achieve an outcome within 14 days of hospital admission. After this down-selection, we were left with a cohort in which 25% of patients had a severe disease or death outcome within 14 days of hospital admission.

The data for each patient included demographics, presence or absence of comorbid conditions, COVID-19 symptoms present at time of admission, and vital signs and common blood laboratory measures per epoch. These features were selected because they are relatively easy to collect and accessible in a broad range of healthcare settings. A full description of the data has been published separately^8^.

**Figure 2:**
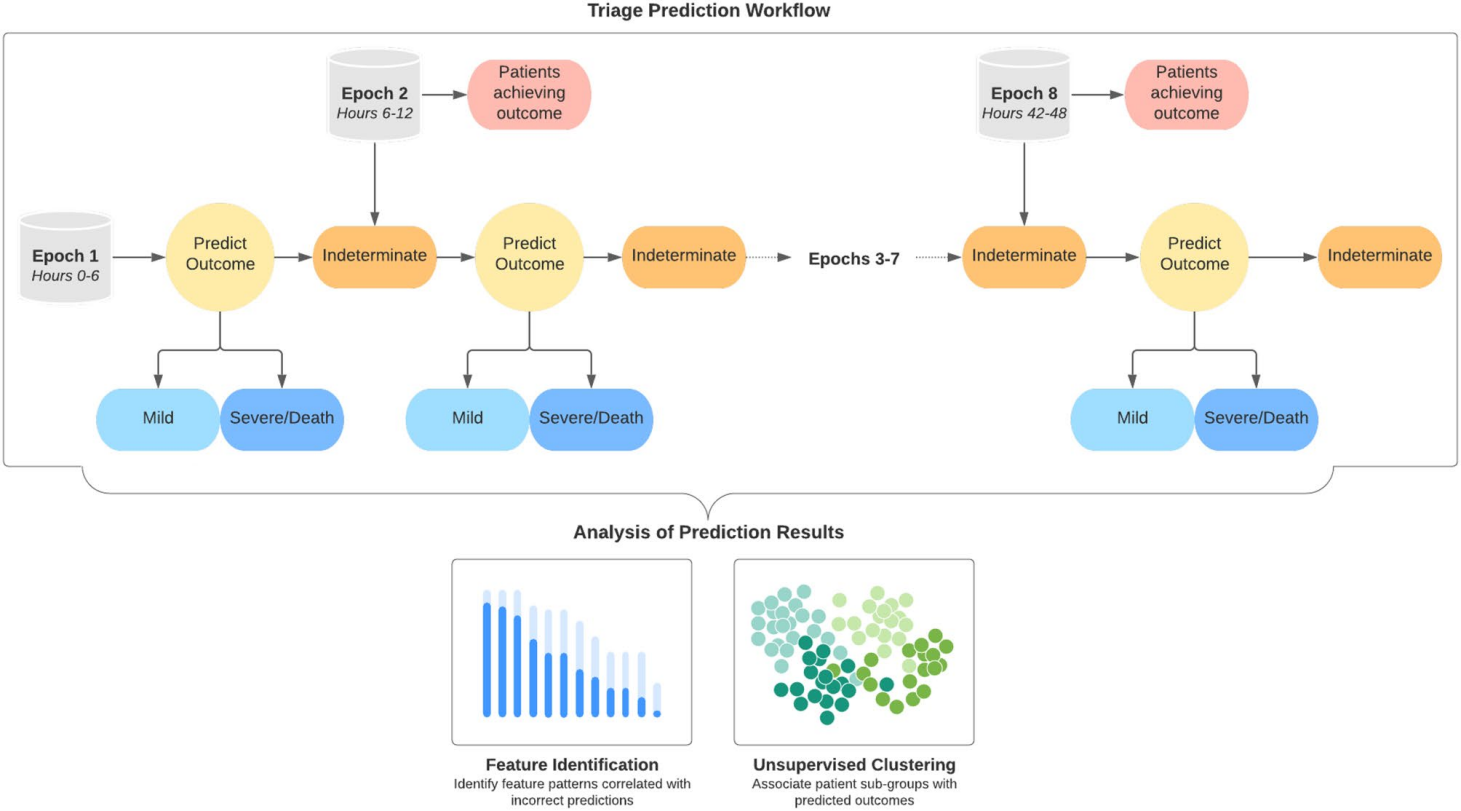
Triaged Prediction Approach. The goal of the triaged prediction approach was to use the most minimal set of data possible to predict a given patient’s outcome and identify potential patient sub-groups based off of prediction patterns. At each epoch of analysis, a classifier was trained and predictions were made using 10-fold cross-validation. Subsequently, patients were classified as either having a mild, severe disease and/or death, or indeterminate 14-day outcome. Patients who achieved an outcome during the epoch were removed from further analysis. After prediction, Kullback-Leibler (KL) divergence was used to identify features that distinguished true positive from false negative predictions and the proportion of errors in each unsupervised cluster were calculated.

### Triaged prediction model

Before designing our own model, we examined the current research in generalizable modeling for COVID-19 patient prediction. Iwendi et al.^25^ successfully applied a random forest classifier to the problem of identifying COVID-19 patients based on symptoms in the outpatient setting. We were informed by this approach in building our models. The supervised model we designed was a triaged prediction approach, where we aimed to use the minimal time window of data possible to determine patient outcomes with high confidence. When an outcome could not be predicted with high confidence, additional data were added in 6-hour increments to augment the dataset available to the classifier. An illustration of the triaged prediction approach workflow can be found in Fig. 2.

At each epoch, a random forest classifier was trained using the data from all patients for whom a high confidence prediction for 14-day outcome was not yet made, using all data up to and including the present epoch. Training and testing was accomplished using 10-fold cross-validation. The classifier could predict a mild outcome, a severe disease/death outcome, or an indeterminate outcome. If a patient had an indeterminate outcome, this meant that the data available for the patient up to and including the current epoch was not sufficient for confidently categorizing them as either severe disease/death or mild. For these patients, the next epoch of data was used and the prediction was re-attempted.

The thresholds for predicting a mild outcome or a severe disease/death outcome, defined as the proportion of decision trees voting for the outcome of interest in the random forest, were 0.9 and 0.5 respectively. These thresholds were chosen after a sweep of positive class thresholds from 0.5 to 0.95 with the goals of reflecting the clinical need for high levels of certainty when declaring that a patient will have a mild outcome, and the desire to limit false negative predictions when determining that a patient will have a severe and/or death outcome after fourteen days. Patients were removed from the epoch-by-epoch analysis pipeline after either (1) a confident prediction could be made about their fourteen-day outcome (regardless of the accuracy of this prediction) or (2) if the patient achieved an outcome of interest in the epoch (severe disease and/or death, or discharge from the hospital with a mild outcome).

Although accurate prediction of patient outcomes was a main goal of the triaged prediction approach model, an equally important goal was to identify patients for whom these prediction methods fail—either by predicting an incorrect outcome or by being unable to render an confident outcome by the end of the 8 epochs of analysis. After predictions were made, we analyzed the characteristics of patients for whom false negative and true positive predictions were made (incorrectly predicting a mild outcome and correctly predicting severe disease or death, respectively). The characteristics of these patients were then used, along with the results of unsupervised clustering of patients, as inspiration for new hypotheses about why particular patients have unpredictable outcomes.

### Unsupervised clustering model

**Figure 3:**
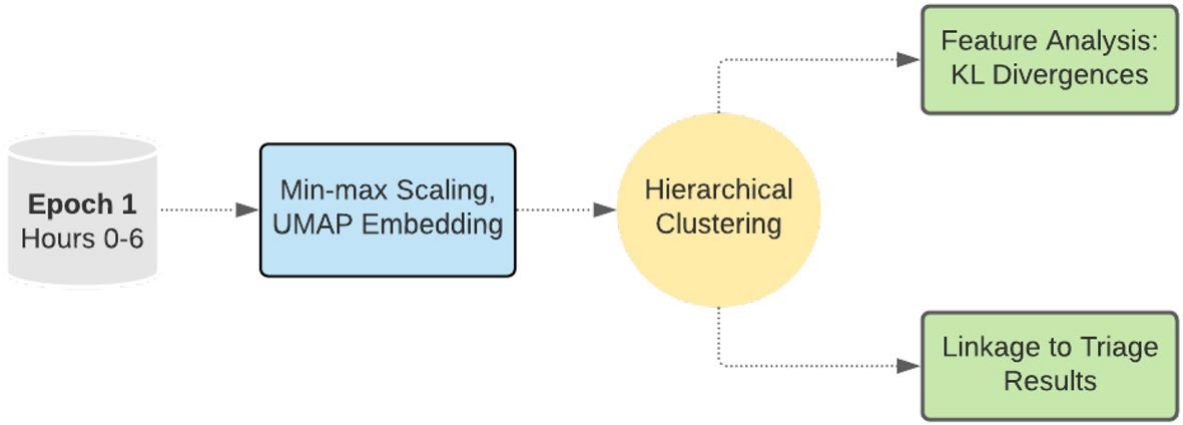
Unsupervised Clustering Approach. Clustering analysis was performed using the first epoch of triaged prediction data. The goal of this complementary model was to find sub-groups of patients at the time of hospital admission. Data were preprocessed using min-max scaling and UMAP embedding to two dimensions. Hierarchical clustering using Ward’s linkage was then applied to generate patient sub-groups. After clustering, the association of cluster membership with patient features and predictions from the triaged prediction model were analyzed towards the goal of further identifying patient sub-groups, hypothesis generation, and assessing the reliability of the triaged prediction approach per sub-group.

Clustering analysis was used to identify patient sub-groups at time of hospital admission with the goal of investigating clinical features that may underpin different disease courses (Fig. 3). The data for performing the unsupervised clustering approach included the first epoch of data as discussed above. Data were min-max normalized, placing features like age, vital signs, and lab test results on the same 0-1 scale as the binary variables encoding comorbidities and symptoms at time of hospital admission. Dimensionality reduction to two dimensions was then performed via Uniform Manifold Approximation and Projection (UMAP) nonlinear embedding^26^ in order to build a low-dimensional representation of our patient cohort. It is important to note that nonlinear embeddings like UMAP can cause issues with distance-based clustering and scoring methods, since densities of data points can be changed in the embedding process. We made attempts to mitigate this problem by enforcing a large number of neighbors in the UMAP embedding, which imposes more global structure in our reduced dataset^26^.

Clustering analysis was performed using hierarchical clustering with Ward’s linkage^27,28^ for a specified 20 clusters. 20 clusters was chosen based on two goals. First, we sought to identify a number of clusters that locally maximized the Calinski-Harabasz (CH) index^29^, the ratio of intercluster to intracluster dispersion. After sweeping over cluster numbers from 1 to 50, we identified that 20 clusters was a local maximum. In addition to use of CH index, we favored a clustering representation that captured a range of disease severity, marked by the proportion of patients in the cluster with an outcome of severe disease or death. Having a broad range of clinical profiles and disease severity was thought to provide the greatest utility in the clinical setting, since patients in each cluster might later be mapped to a prognosis and action plan.

After clustering, cluster labels were mapped to the original data for analysis of sub-group feature differences. We then analyzed the cluster feature distributions to find defining characteristics of each cluster. To find features that were the most powerful for differentiating clusters, symmetric Kullback-Leibler (KL) divergence^30^ calculations were performed on the feature distributions for each pair of identified clusters. The pairwise KL divergence measures were then summed to yield a total KL divergence for each feature. Features that had higher KL divergence information-gain values were then interpreted as being more important for separating clusters overall.

In summary, clustering was used to generate patient sub-groups based on information present early in a patient’s hospital stay. We analyzed the composition of each cluster and the association between sub-group classification and outcome. KL divergence was used to identify features that may typically drive differences in patient sub-groups. These features can be interpreted as being more important for identifying patient sub-groups, potentially selecting risk factors that separate patients into low-risk or high-risk subgroups within the population.

## Results

### Triaged prediction

Of the 1175 patients analyzed, 156 patients continued to have indeterminate predictions after eight epochs (48 hours) of analysis. A confusion matrix of prediction results by epoch of analysis with the number of true positive, false positive, true negative, and false negative predictions, and number of indeterminate and patients removed from analysis for achieving an outcome of interest before a prediction could be made, can be found in Table 1. Across all epochs, the false negative rate is low and the indeterminate rate is high—a product of our design choice to avoid making the erroneous and harmful prediction that a particular patient will have a positive outcome when in actuality, their outcome will be poor. This algorithm design choice mimics the watchful waiting approach that a clinician might take when faced with a patient for whom they are unsure will have a positive outcome, and may lead to more trusted and reliable predictions.

**Table 1:** Prediction results for each epoch of analysis. Guided by clinical input, the triaged prediction algorithm was designed to predict outcomes with a low false negative rate at the expense of a higher indeterminate prediction rate. Patients were removed from the prediction process if they achieved an outcome of interest. The trade-off between false negative and indeterminate prediction results in 156 patients for whom a prediction could not be made within 8 epochs of analysis. For different clinical use-cases, the thresholds for making a positive or negative prediction could be adjusted.

Epoch	TP	FP	TN	FN	Indeterminate	Removed for achieving outcome
1	75	36	243	7	814	0
2	16	15	55	7	622	99
3	14	6	32	2	495	73
4	2	2	18	5	413	55
5	0	1	16	1	330	65
6	2	3	12	1	234	78
7	0	2	10	2	195	25
8	2	0	0	2	156	25

The mean accuracy for prediction of 14-day outcome across the six-hour epochs (10-fold cross validation for each epoch) was 0.83 ($\sigma =0.08$), and the mean Matthew’s Correlation Coefficient (MCC) was 0.24 ($\sigma =0.29$). Accuracy, AUC, and MCC per epoch of analysis can be found in Table 2. Of particular note is the high variability in MCC, a result of the predictions becoming more error-prone in later epochs of analysis. This is expected behavior due to the smaller numbers of patients eligible for analysis in later epochs. Additionally, later epochs of analysis may have more difficult patients to predict because the only patients present for prediction in these epochs are ones for whom a prediction could not be made previously.

**Table 2:** Mean metrics for each fold of 10-fold cross-validation over the 8 6-hour epochs under study (48 hours total). After 3 epochs, performance drops as marked by the stark decrease in the Matthew’s Correlation Coefficient Metric, illustrating the difficulty of predicting outcomes for the subset of individuals that have indeterminate predictions in the preceding epochs.

Epoch	Accuracy	AUC	MCC
1	0.88	0.90	0.71
2	0.78	0.81	0.39
3	0.85	0.91	0.58
4	0.77	0.45	0.17
5	0.90	0.50	0.00
6	0.83	0.75	0.15
7	0.67	0.00	-0.10
8	0.92	0.75	0.05

For each epoch, the mean impurity-based random forest feature importances were calculated using the Scikit-Learn package^28^ across the 10 folds of cross validation. The most important feature in each epoch was the SpO_2_/FiO_2_ ratio within the epoch. The SpO_2_/FiO_2_ ratio from the previous epoch was highly important for classification as well, ranking as the 2nd most important feature in epochs 2–7 and the 4th most important feature in epoch 8. The relative importance of the SpO_2_/FiO_2_ ratio for predicting outcomes are consistent with prior findings of its importance in predicting acute respiratory distress syndrome^31^.

The triaged classification system is imperfect at predicting 14-day outcome, but has possible utility for uncovering sub-groups of patients who have characteristics making them prone to mis-classification or indeterminate outcome. To investigate the differences between those patients for whom a true positive outcome prediction (i.e., the model correctly predicted that the patient developed severe disease or death within the first 14 days of hospital admission) and false negative prediction (i.e., the model incorrectly predicted that a patient had a mild fourteen-day outcome) were made, we analyzed which features were most informative for separating out these patients in the first two epochs of analysis via KL divergence^30^.

The first two epochs were the only epochs out of the eight with more than 5 patients having true positive and false negative prediction outcomes. Therefore, we analyzed these two epochs for the features that distinguish the true positive and false negative patient groups from each other. Through KL divergence calculations, we found that the most important features for distinguishing the false negative and true positive patient groups were SpO_2_/FiO_2_ ratio, albumin and GFR lab test results, and the oxygen device that the patient used. For predictions made in epoch 2, the the values of SpO_2_/FiO_2_, albumin and GFR within epoch 2, as well as from epoch 1, were found to be important for distinguishing between the true positive and false negative groups. The differences in feature values between the groups are illustrated in Tables 3 and 4. Across epochs 1 and 2, patients for whom a false negative prediction was made appear to have significantly higher SpO2/FiO2 ratios, as well as GFR and albumin test results that may fall into normal range during the first epoch of analysis.

**Table 3:** Key feature differences between TP and FN groups predicted during epoch 1. SpO_2_/FiO_2_, albumin, estimated glomerular filtration rate (GFR) test results, and mode of oxygen delivery during epoch 1 were found to be the most important for distinguishing patients for whom a false negative and true positive prediction was made at this stage of analysis. Feature importance was calculated via KL divergence and statistical testing for differences of means for laboratory and vital signs were conducted via ANOVA (* p < 0.05, ** p < 0.01, *** p < 0.001).

	FN	TP
**Laboratory and citals (Mean Values)**		
SpO_2_/FiO_2_ ***	472.9	202.7
Albumin *	3.7	3.3
GFR **	99.6	50.5
**Oxygen device (N patients)**		
Room air	7	3
Nasal cannula	0	45
Mask	0	27

**Table 4:** Key feature differences between TP and FN groups predicted during epoch 2. SpO_2_/FiO_2_ measured in epochs 1 and 2, the type of oxygen delivery patients received in epochs 1 and 2, as well as the albumin and estimated glomerular filtration rate (GFR) test results from epoch 2 were found to be the most important for distinguishing patients for whom a false negative and true positive prediction was made at this stage of analysis. Feature importance was calculated via KL divergence and statistical testing for differences of means for laboratory and vital signs were conducted via ANOVA (* p < 0.05, ** p < 0.01, *** p < 0.001).

	FN	TP
**Laboratory and vitals (Mean Values)**		
SpO_2_/FiO_2_ (From Epoch 1) ***	454.3	252.2
SpO_2_/FiO_2_ (In Current Epoch) ***	477.9	231.5
Albumin (From Epoch 1) *	3.8	3.6
GFR (From Epoch 1) * *	84.4	46.8
**Oxygen device in epoch 1 (N patients)**		
Room air	6	0
Nasal cannula	1	16
**Oxygen device in epoch 2 (N patients)**		
Room air	7	0
Nasal cannula	0	13
Mask	0	3

Overall, the patients for whom a false negative prediction was made had more mild symptoms during the first 2 epochs (12 hours) of analysis. The set of patients for whom a false negative error is made may represent a particularly interesting sub-group of patients for further investigation. Our unsupervised clustering approach is one method to investigate those features that are associated with an increased risk of erroneous or indeterminate outcomes.

**Figure 4:**
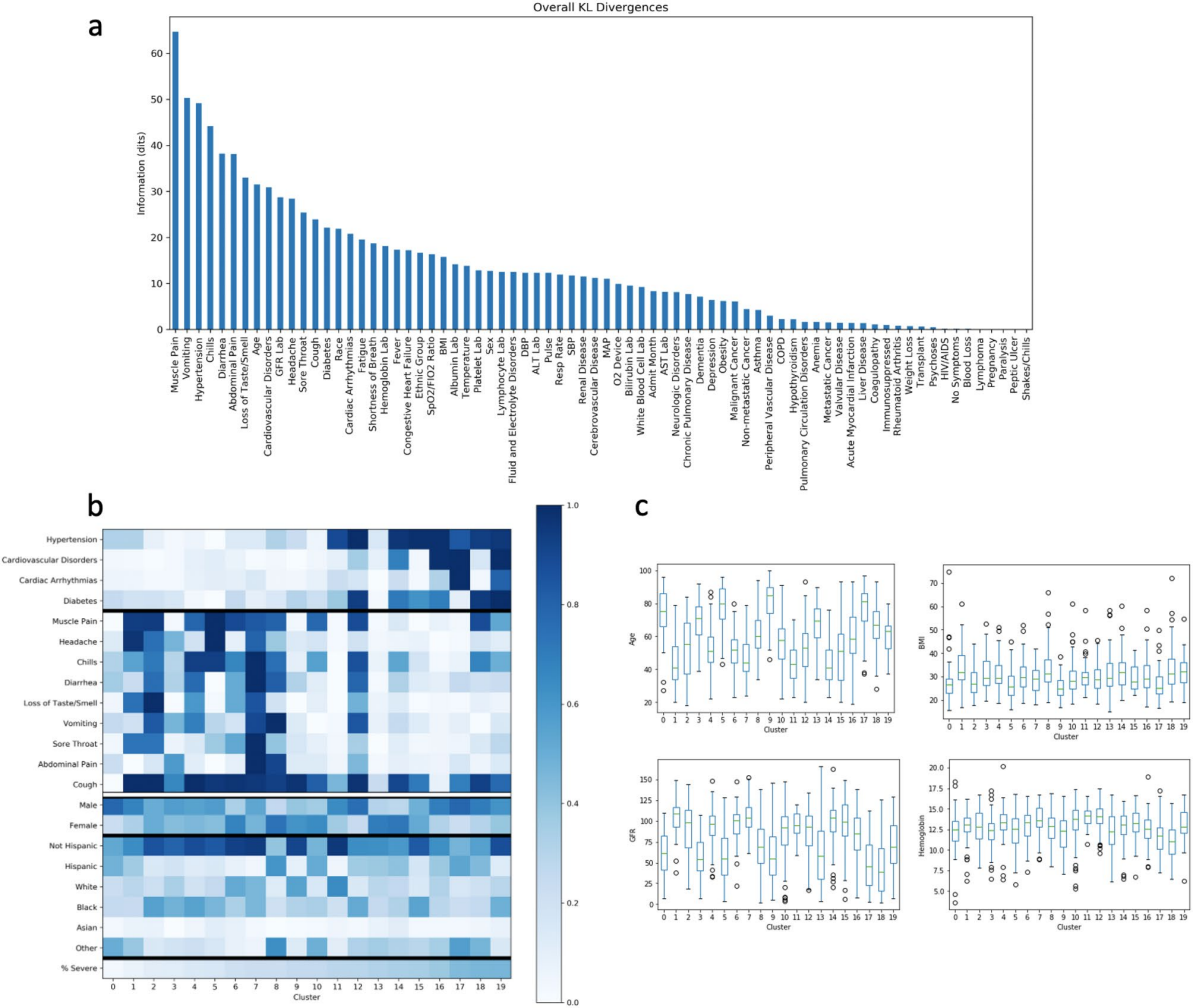
Feature differences among clusters. (**a**) Cluster-wise symmetric KL Divergences, sorted by information gain. Taller bars indicate that the feature is more important for distinguishing between unsupervised clusters. (**b**) Heatmaps from top 20 KL divergences for binary features (comorbidities, symptoms), sorted by KL divergence values. Plotted values are proportions of present features for a given cluster, ranging from 0 to 100%. Proportion of patients per cluster with particular demographic attributes are also plotted for reference. Clusters are sorted by the proportion of patients with severe disease/death outcomes within the cluster (bottom row). (**c**) Boxplots for age, BMI, GFR, and hemoglobin values per cluster. These data are presented as boxplots instead of in the heatmap fashion in subplot (**b**) given the continuous nature of the data.

### Unsupervised clustering

Our clustering approach found patterns in patient sub-groups that may indicate potential clinically-relevant differences in disease progression with respect to a given patient’s disease presentation at admission. By design, the 20 clusters identified exhibit a wide range of disease outcomes: each cluster is comprised of anywhere from 2% to 46% of patients achieving the severe disease/death outcome, shown in Fig. 4b. Via KL divergence, specific features were identified that drive separability of the 20 clusters. These features fall into the following categories: presence or absence of specific admission symptoms and comorbidities, age, race, ethnic group, and lab test results. Admission symptoms found to be important for separating clusters included muscle pain, headache, chills, diarrhea, loss of taste/smell, vomiting, sore throat, abdominal pain, and cough. Interestingly, this list includes all available symptoms except for fever, fatigue, shortness of breath, and “no symptoms” (kept separate to differentiate whether a patient affirmed that they had no COVID-19 symptoms, versus whether no symptoms were entered by the provider). Specific comorbidities important for separating clusters included hypertension, cardiovascular disease, cardiac arrhythmia, and diabetes. For visualization purposes, we separated out binary variables, which are expressed as proportion of individuals having the feature within the cluster, from continuous variables, expressed as the distribution of the feature within the cluster. Continuous features found to be important included age, BMI, GFR, and hemoglobin lab tests. A visualization of feature KL divergences can be found in Fig. 4a.

A visualization of categorical and continuous variables and their relationship to cluster membership and proportion of individuals achieving a severe and/or death outcome can be found in Fig. 4b and c. Clusters with a higher proportion of severe outcomes tended to have a higher proportion of comorbidites and lower proportion of reported symptoms when compared to clusters with a higher proportion of mild outcomes. However, our analysis finds more fine-grained sub-groups of patients for which this general trend is not the case. For example, cluster 13 has a relatively high incidence (33%) of severe outcomes but low proportions of both comorbidities and symptoms. This group of patients does, however, tend to be higher in age than other patient clusters, which could be a driving force for worse outcomes. Clusters 7 and 11 also have higher and lower incidences of symptoms, respectively, when compared to neighboring clusters. Patient age, previously identified by researchers to drive patient outcomes^32^ does not appear here to have a clear relationship with cluster membership (and thus a relationship with proportion of individuals having a severe disease/death outcome) when taken in isolation.

**Figure 5:**
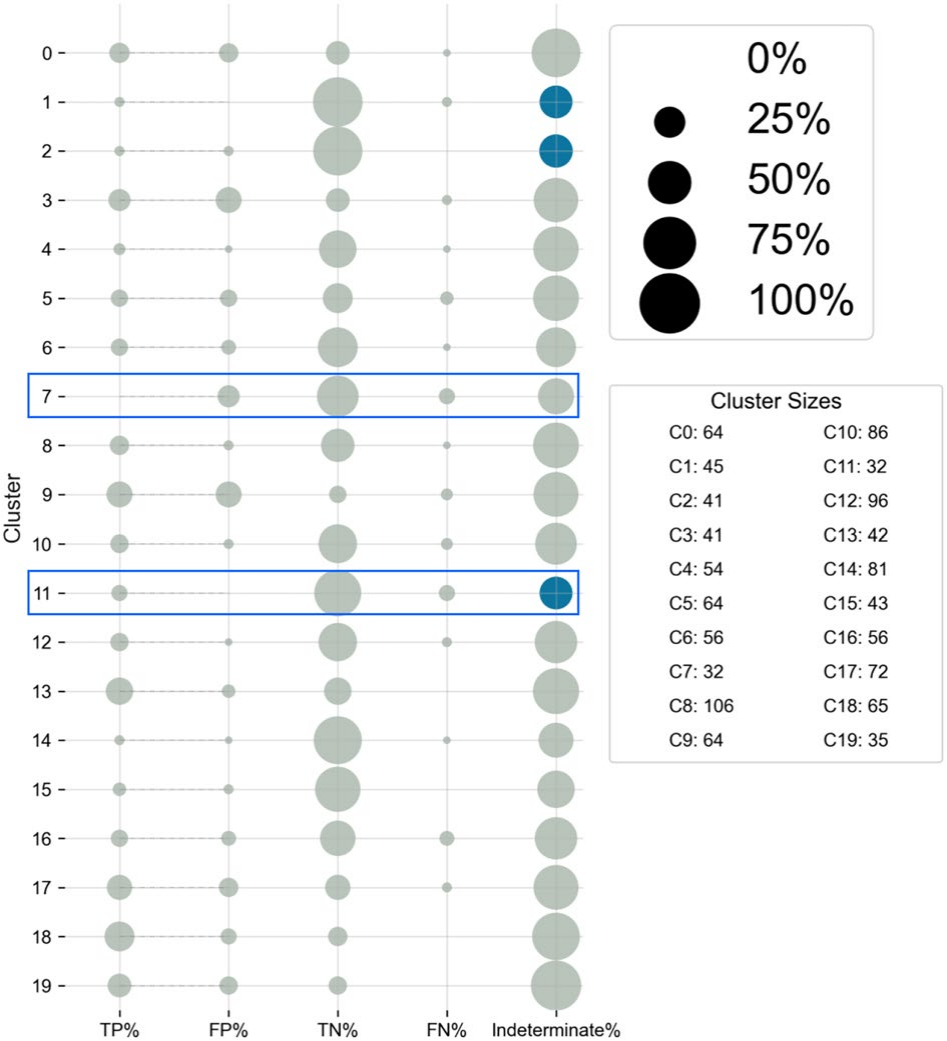
Triaged classification results by unsupervised cluster. The cluster membership of patients was cross-referenced with the final prediction result obtained, connecting the patient presentation within the first epoch of analysis with the final prediction outcome. Sizes of circles represent the proportion of patients within the cluster with the specified classification results. Blue circles in the “Indeterminate” column indicate that less than 30% of patients still had an indeterminate classification after 48 hours of the triaged prediction approach. Boxes indicate clusters within which more than 5% of patients had a false negative prediction result. Visualizing the data in this manner aids in finding sub-groups of patients who are particularly well-suited for the triaged classification approach and drives discussion about factors potentially influencing classification results.

Finally, we analyzed patterns between cluster membership and triaged prediction results, examining the proportion of patients in each cluster for whom a true positive, true negative, false positive, false negative, or indeterminate prediction was made. The results of this analysis can be found in Fig. 5. Clusters 1, 2, and 11 all have less than 30% of the cluster remaining with an indeterminate prediction after 48 hours, denoted by a blue colored circle in the figure. For patients in these clusters in particular, the triaging approach as described seems particularly well-suited for determining patient outcomes with certainty. However, that high certainty does not always map to accurate predictions.

There are two clusters, indicated by blue boxes, for which greater than 5% of patients had a false negative classification result: clusters 7 and 11. Cross-referencing these clusters with the symptom information displayed in Fig. 4 b, we found that these sub-groups with high false negative classifications also were noted to be exceptions to the general trends seen in other sub-groups, where high incidence of symptoms is related to better outcome. In particular, cluster 7 has high incidence of all symptoms found to be important for differentiating sub-groups, while cluster 11 has very few patients with incidence of these symptoms. For patients in these clusters, additional features not used in our triaging approach are likely necessary to make accurate predictions about disease outcome. Patients hypothesized to belong to these clusters should be treated with extra care when predicting outcomes, as they may appear to be on track for a more mild outcome early in their disease course, but instead achieve a severe and/or death outcome.

Combining the results from the supervised triaged prediction algorithm with unsupervised clustering results aids understanding of which clusters of patients may have outcomes classified reliably, and for which patient groups extra caution (or further modeling) must be taken. Understanding which patients are and are not served by the algorithm is especially important for advancing equity and reducing bias in machine learning-assisted patient care.

## Discussion

In this work, we have shown how two complementary data science models were used, together with clinical insight, to identify patterns in patient characteristics and clinical features that may lead to differences in COVID-19 disease presentation and outcomes. Through our triaged prediction model, we grounded our search for patient sub-groups in predicting whether patients will have mild or severe disease/death outcomes. In our clustering model, we searched for patterns in patients as they first presented to the hospital, with a particular focus on investigating how characteristics of these unsupervised groupings of individuals might contribute to the ease or difficulty with which an outcome can be predicted. In addition to driving hypothesis generation, analyzing triaged prediction results in combination with the unsupervised clustering approach showed that the classifier is not equally reliable in all patient sub-groups. This provides clincians with a lens to contextualize classifier results based on underlying patient characteristics and knowledge to use the predictions appropriately in a care setting. Towards the goal of equitable machine learning-assisted patient care, understanding that the predictive algorithm is limited for particular sub-groups of patients is important and is an area of future work.

The unsupervised clustering results indicate that clinically-relevant patient sub-groups may be found in COVID-19 using readily-available patient data. Importantly, these sub-groups are characterized by the complex interactions between variables in patient health records. These sub-groups have different characteristics at time of admission and different proportions of severe disease/death outcomes. It may be more difficult to accurately predict outcomes for some sub-groups of patients, as shown via the combination of triaged prediction results and the unsupervised clustering analysis. Although the exact reasons for the differences in these sub-groups are beyond the scope of the present study, analyses such as these may lead to the generation of clinical hypotheses that are valuable in public health emergencies.

Our clustering analysis produced discussion across our collaborative clinical and data science team as we sought to explain why the clustering results indicated that, in general, clusters with a higher percentage of severe patients had a lower incidence of symptoms like diarrhea, loss of taste or smell, vomiting, sore throat, and abdominal pain. One hypothesis is that these patients enter the hospital when they are so severely ill that these symptoms remain undocumented, either because a patient cannot express these symptoms themselves, or because a patient is quickly attended to before this information is added into the medical record. The disease profiles of the milder patients with high prevalence of symptoms may also indicate more active immune systems, which cause worse symptoms early in a patient’s disease course but a less severe outcome overall. These hypotheses could be used to investigate clinical care practices or some underlying factor in the disease model not yet uncovered.

Some other areas for future investigation are highlighted by this work, such as why the patients with false negative predictions in the first two epochs seemed to have more positive clinical profiles during the first two epochs of analysis when compared to their counterparts with true positive-predicted outcomes, or how the addition of other variables, such as socioeconomic status and patient location, impact sub-group identification. Of potential interest to a clinical care provider might be why some patients continue to have an indeterminate prediction even after 48 hours of analysis, and how to accurately treat these patients for the best odds of survival. While this work cannot find answers to these questions, it is this style of modeling that may aid clinical research teams in partnership with data science teams in identifying clinical patterns that were previously not observed.

Like all models, our implementation comes with limitations. We cannot infer a causal relationship between the features of interest that separate patient sub-groups, since our models infer statistical dependencies only. There are limitations to the dataset used as well; we did not have access to the time since symptom onset, which might impact results because some patients may present to the hospital at different times in their COVID-19 disease course for different reasons. Additionally, although we emphasize the generalizability of our overarching approach of pairing supervised machine learning with unsupervised clustering for hypothesis generation and data exploration, our specific model implementations may not perform well on all disease models. Choosing appropriate models and then tuning models to a different disease or sub-group identification problem will require both clinical and data science expertise. We note that our dataset was drawn from early admissions during the COVID-19 pandemic and due to the emergence of different disease variants, vaccines, and human behaviors, these results may not generalize to contemporary cases. In the current study, the data were carefully collected, quality controlled, and stored in an easily-accessible format, which surely contributed to our success in this work. Approaches like ours, which draw on collaborative data-collection efforts and standardization protocols, have great potential for improving treatment efficacy and enabling clinicians to make different decisions when allocating limited resources and clinical care. This is a powerful argument for data standardization and co-location of compute infrastructure with clinical data^23,33^, *before* the next public health crisis arises.

While others have reported similar methods employing both unsupervised clustering and supervised prediction approaches, our work differs in its goal: to provide a paradigm for hypothesis generation. For example, clustering prior to classification has been shown to simplify predictive models of persistent high health care utilization without costs to performance^34^. Additionally, applying clustering prior to regression was found to improve regression accuracy in predicting length of stay and mortality in the MIMIC II dataset^35^. Clustering and federated learning have also been combined to predict patient outcomes accurately, in a manner that limits data sharing between healthcare institutions and protects data privacy^36^. Finally, clustering has been used side-by-side with other statistical techniques, such as grouping oncology patient health trajectories after identifying statistically significant covariates for health decline^37^. Comparing a combined prediction and clustering approach to other approaches using only one or the other may be an area for future work.

One important contribution of this work is our paradigm for combining clinical and data science knowledge together to build models that drive hypothesis generation. In our specific implementation, we show the power of partnership between data scientists and clinical experts when working to create new precision medicine tools and generating new avenues for investigation. Our two-pronged triaged outcome prediction and data-driven sub-population discovery procedure may be easily extended to other disease models and domains. In this way, data science can be employed as a tool that “points clinical researchers to the *right* haystack” for further exploration and analysis. This is an especially useful approach in the context of emerging disease threats where existing knowledge is limited. In the future, precision modeling will likely continue to provide ground-breaking predictive modeling and sub-population discovery insights. These approaches, additionally, may provide an interface between emerging data science and clinical judgement. In this way, the data science underpinning precision medicine may serve as a hypothesis-generation engine, which can speed up clinical progress, and ultimately provide better care to patients especially in the face of rapidly changing clinical landscapes.

## Data Availability

The data used were part of JH-CROWN: The COVID Precision Medicine Analytics Platform Registry^21^ and some patients analyzed here have been included in previous descriptions of the cohort^8,17–19,22–24^. Data and analysis code will be made available upon researcher request as allowable by the terms of the registry and the Johns Hopkins Medicine Institutional Review Board (IRB). This work was approved under IRB00250975.
